# Supplementary material for: Ductile Copolyesters Prepared Using Succinic Acid, 1,4-Butanediol, and Bis(2-hydroxyethyl) Terephthalate with Minimizing Generation of Tetrahydrofuran
Source: Polymers (Basel). 2024 Feb 14;16(4):519. doi: 10.3390/polym16040519 (PMC10891720; doi:10.3390/polym16040519)

Supporting Information

Copolyesters Prepared Using Succinic Acid, 1,4-Butanediol, and Bis(2-hydroxyethyl) Terephthalate

Sang Uk Park, Hyeon Jeong Seo, Yeong Hyun Seo, Ju Yong Park, Hyunjin Kim, Woo Yeon Cho, Pyung Cheon Lee, and Bun Yeoul Lee*

Department of Molecular Science and Technology, Ajou University, Suwon 16499, South Korea; gkdl3094@ajou.ac.kr (S.U.P.); hjseo@ajou.ac.kr (H.J.S.); tdg0730@ajou.ac.kr (Y.H.S.); pjy3741@ajou.ac.kr (J.Y.P.); khj610@ajou.ac.kr (H.J.K.); wycho418@ajou.ac.kr (W.Y.C.); pclee@ajou.ac.kr; (P.C.L.)

* Correspondence: bunyeoul@ajou.ac.kr (B.Y.L.); Tel.: +82-31-219-1844

Figure S1. ^{31}P NMR spectra of copolyesters prepared with varying H_3PO_4 feed quantities (1.0, 2.0, 3.0 mol% per SA).

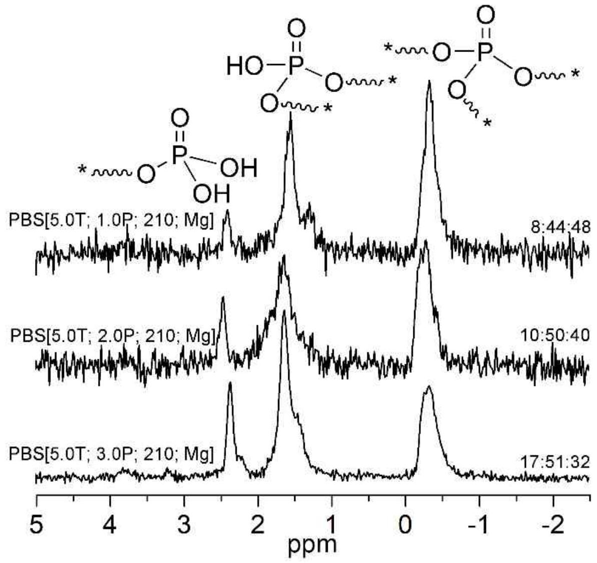

Figure S2. GPC curves for copolyesters prepared with a) variation of temperatures (190, 200, 210, and 220 °C), b) varying BHET feed quantities (0, 5.0, 10, 15 mol% per SA), and c) varying H_3PO_4 feed quantities (1.0, 2.0 mol% per SA).

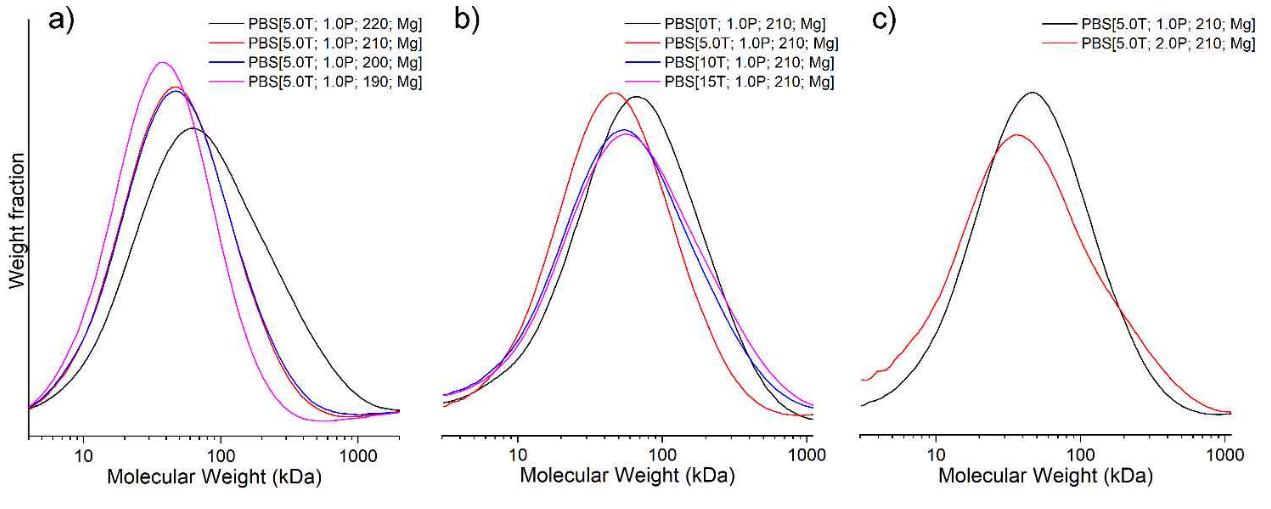

Figure S3. a) Images illustrating reactor fouling during the esterification of BD, SA, and dimethyl terephthalate, contrasted with b) image depicting the cleanliness observed in the corresponding reaction involving BD, SA, and BHET.

a) b)

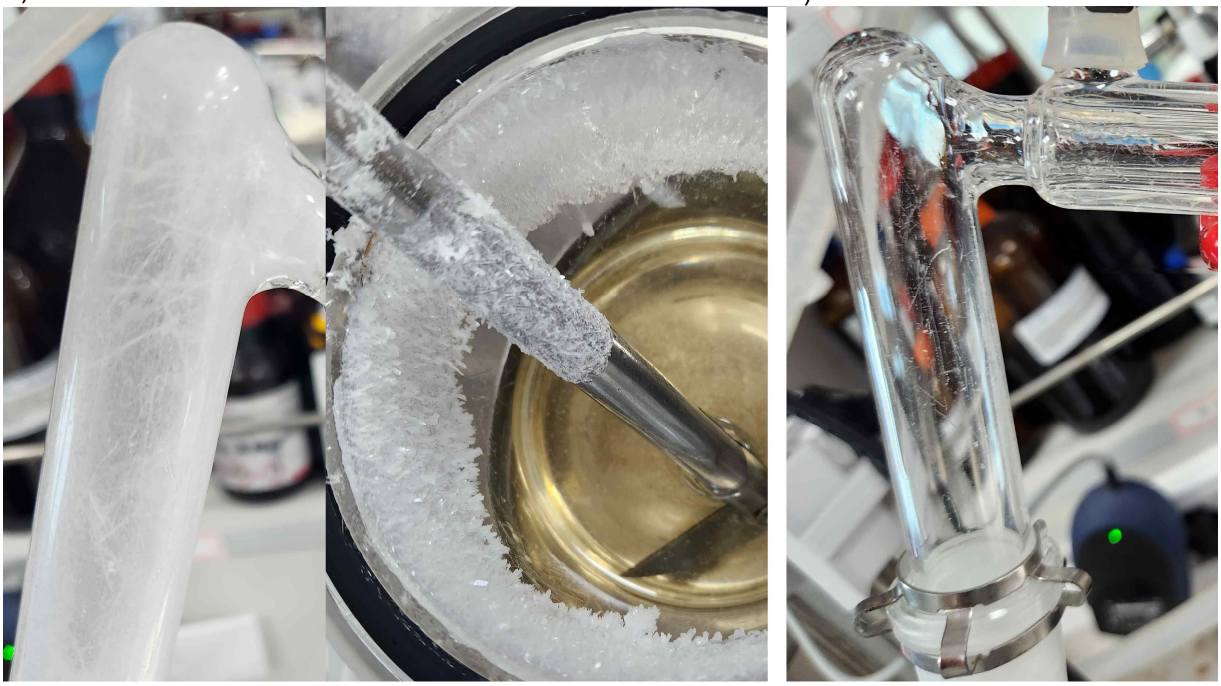

Figure S4. Temperature dependence of a) tanδ, b) storage modulus (E'), c) loss modulus (E") curves from DMA runs illustrated for PBS[xT; Ti; 230] (x = 0, 5.0, 10, 15, 20) series.

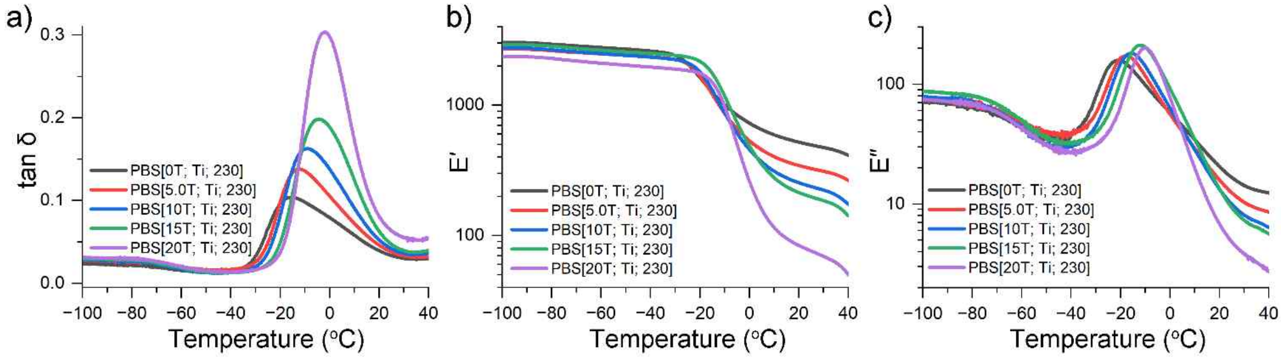

Figure S5. Tensile curves for copolyesters prepared with a) variation of temperatures (200, 210, and 220 °C), b) varying H_3PO_4 feed quantities (1.0, 2.0, 3.0 mol% per SA), and c) varying the divalent metal species (Mg, Zn, Mn).

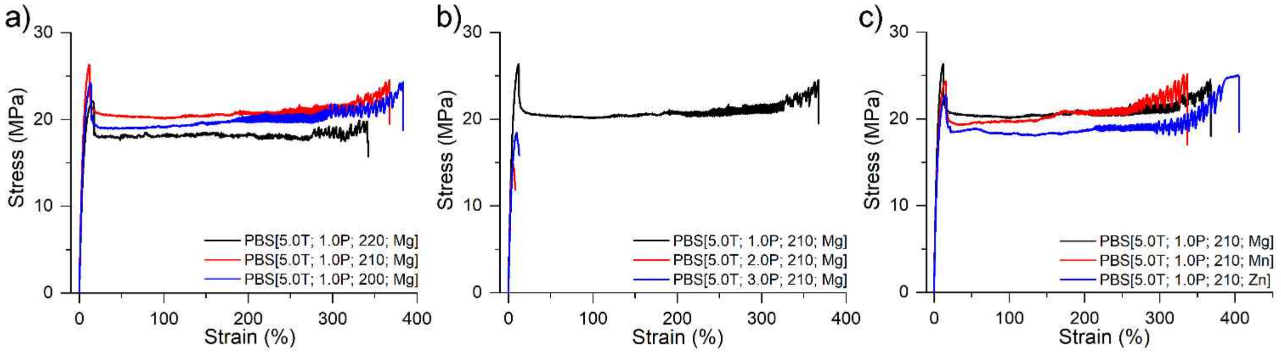

Figure S6. Comparison of dynamic moduli vs. angular frequency between a) PBS[0T; 1.0P; 210; Mg] and PBS[0T; Ti; 230], b) PBS[5T; 1.0P; 210; Mg] and PBS[5T; Ti; 230], c) PBS[15T; 1.0P; 210; Mg] and PBS[15T; Ti; 230], d) PBS[20T; 1.0P; 210; Mg] and PBS[20T; Ti; 230].

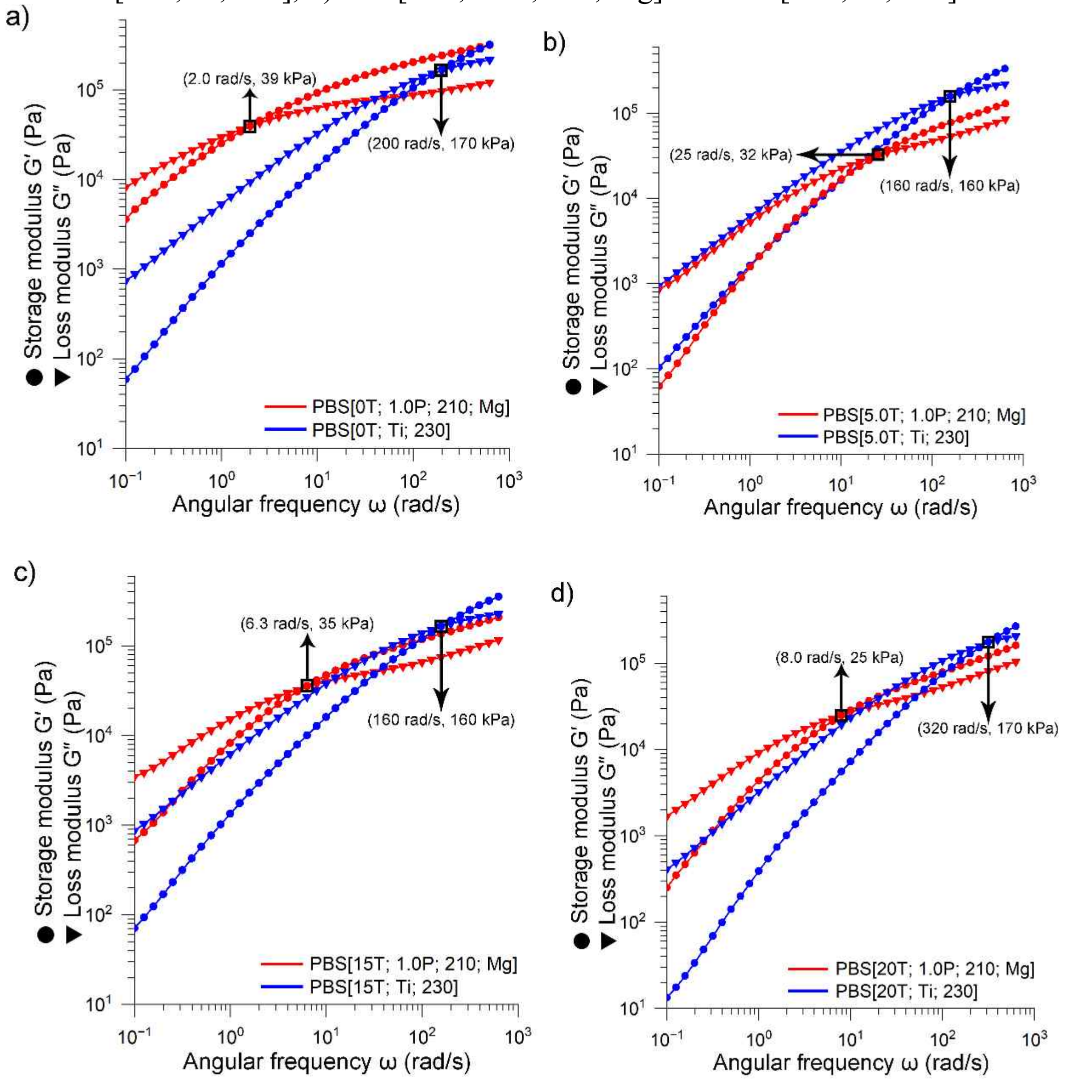

Supplement: Supplementary file 1 [file polymers-16-00519-s001.zip › polymers-2846826-supplementary.pdf]
